# Do Self-Compassion Factors Affect Value-Based Behavior Promotion for Adolescents? The Context of Behavioral Activation

**DOI:** 10.3389/fpsyg.2021.566181

**Published:** 2021-10-14

**Authors:** Koki Takagaki, Satoshi Yokoyama, Kohei Kambara

**Affiliations:** ^1^Health Service Center, Hiroshima University, Higashi-Hiroshima, Japan; ^2^Department of Psychiatry and Neurosciences, Hiroshima University, Hiroshima, Japan; ^3^Graduate School of Education, Hiroshima University, Higashi-Hiroshima, Japan

**Keywords:** adolescence, value-based behavior, self-compassion, behavioral activation, behavioral characteristics

## Abstract

Behavioral activation has been shown to engender improvement of depressive symptoms. Behavioral activation specifically addresses behavioral patterns that maintain depressive symptoms and increases activation of rewarding behaviors based on a person’s own values. Nevertheless, behaving according to those values can be difficult. Consequently, discussing reduction in barriers to activation of value-based behaviors is important. Self-compassion might promote value-based behavior when a person confronts some obstacle. We evaluated differences in self-compassion and behavioral characteristics at high and low levels of value-based behaviors. Furthermore, we examined relations of self-compassion to value-based behaviors while considering activation, avoidance, and positive reinforcement in the context of behavioral activation. All 800 university student participants (415 women, 385 men; mean age=20.47years; age range 18–24years) in this study completed assessments. Descriptive statistics, MANOVA, and stepwise regression analysis relations elucidated their value-based behavior, self-compassion, and behavioral characteristics. Results suggest that participants who behaved according to values had high levels of self-kindness, common humanity, and mindfulness. Results of stepwise regression analyses suggest as a new finding that positive components of self-compassion (self-kindness, common humanity, and mindfulness) are related to value-based behaviors even when behavioral factors are considered in the context of behavioral activation.

## Introduction

Depression, which has been estimated as affecting 15–20% of university students (Benton et al., 2003), can adversely affect academic performance and social activity (Balazs et al., 2013). Behavioral activation is an empirically supported intervention for depression (Ekers et al., 2014; Richards et al., 2016). The Martell behavioral activation model (Martell et al., 2001) includes specific emphasis of the role of avoidant behaviors. The model is consistent with traditional behavioral models: Behavioral activation modifies a person’s environment through behavioral change, which in turn increases access to positively reinforcing events and behaviors based on a person’s own values (Manos et al., 2010). Therefore, increasing positive reinforcement contingencies based on a person’s own values is important.

Behavioral activation specifically assesses behavioral patterns that maintain depressive symptoms and which increase the activation of rewarding behaviors based on a person’s own values (Kanter et al., 2009; Martell et al., 2010; Takagaki et al., 2016a). Values are defined as freely chosen, verbally constructed consequences of ongoing, dynamic, evolving patterns of activity, which establish predominant reinforcers for that activity (Wilson and DuFrene, 2009). They are intrinsic in engagement in the valued behavioral pattern itself (Wilson and DuFrene, 2009). As one illustration of behavioral activation, participants conducted behavioral experiments to modify avoidant behavior and to increase value-based behaviors (e.g., Kanter et al., 2009; Takagaki et al., 2018). Engaging in value-based activities invariably triggers some psychological characteristics that can otherwise act as barriers to action when the content is negative (Hayes et al., 2011). Engaging in the behavior might become automatically reinforcing simply through linkage with long-term valued consequences (Kanter et al., 2009). This linkage might then sustain behavior in the face of extremely strong competing short-term contingencies (Kanter et al., 2009). For example, people who value competition in marathons might continue to run despite a multitude of aversive stimuli that would discourage and deter running in other people (Kanter et al., 2009). Behaving in accordance with values can be stressful and difficult (Bear and Nagy, 2017), but a report of an earlier study described that value-based behaviors are positively related to well-being (Smout et al., 2014; Zessin et al., 2015). Therefore, it is necessary to discuss factors that promote value-based behaviors in people, even when they are facing some obstacles.

Results of an earlier study suggest that, when confronting a threat of future negative events, self-compassionate people are more likely to address them proactively (Allen and Leary, 2010). Reports of earlier studies investigating relations between self-compassion and behavior have described a positive relation with positive components of self-compassion (self-kindness, common humanity, and mindfulness) and health behavior (Gedik, 2019; Holden et al., 2020). Moreover, self-compassion is negatively related to rumination (Neff et al., 2007). Self-compassion involves being touched by and being open to one’s own suffering, not avoiding or disconnecting from it, generating a desire to alleviate one’s suffering and to heal oneself with kindness (Neff, 2003). People who are self-compassionate are more likely to accept responsibility for their mistakes and failures than those who are less self-compassionate (Leary et al., 2007). Furthermore, treating oneself kindly despite one’s own problems and failures allows a person who has a high degree of self-compassion to accept responsibility and to continue with other activities (Allen and Leary, 2010). Increasing positive reinforcement contingencies based on one’s own values is important for behavioral activation. Behaving in accordance with one’s own values can be stressful and difficult (Bear and Nagy, 2017), but self-compassion may have some role in promoting value-based behavior when one faces obstacles.

Few reports have described examinations of relations among value-based behaviors and self-compassion in a behavioral activation context. Martell’s behavioral activation model (Martell et al., 2001) includes specific emphasis of the roles of avoidant behaviors. In that model, behavioral activation modifies a person’s environment through behavioral change, which in turn increases access to positively reinforcing events and activities (Manos et al., 2010). Therefore, activation, avoidance, and positive reinforcement are regarded as important factors for behavioral activation. In the context of behavioral activation, when considering etiology and therapeutic mechanisms, studies mainly address factors such as activation, avoidance, positive reinforcement, and depressive symptoms (e.g., Manos et al., 2010; Takagaki et al., 2014, 2016b). However, characteristics of self-compassion and main factors of behavioral activation must be considered because self-compassion might play a role in promoting value-based behavior when confronting obstacles.

Debate persists about scoring of the self-compassion scale. One earlier study (Neff et al., 2019) supported the use of a total score or six subscale scores. Based on that earlier study, we used six factors to examine details of self-compassion characteristics. The features specifically addressed by the degree of the value-based behaviors can be clarified if those behavioral characteristics and self-compassion differ depending on the frequency of the value-based behaviors. Therefore, we strove to ascertain whether differences exist in behavioral characteristics and self-compassion at high and low levels of value-based behaviors. Additionally, we examined whether self-compassion is related to value-based behaviors considering activation, avoidance, and positive reinforcement.

We assessed the following hypotheses in this study. Differences exist in characteristics of self-compassion depending on the frequency of value-based behaviors. Self-compassion is related to value-based behaviors considering activation, avoidance, and positive reinforcement.

## Materials and Methods

### Participants and Procedures

The study participants, 800 Japanese undergraduate university students (415 women, 385 men; mean age=20.47, 18–24years, SD=1.39), were recruited from Rakuten Insight, Inc., a market research company that conducts surveys using the Internet. Many undergraduate students in Japan belong to Rakuten Insight, Inc. We asked undergraduate students to participate in this survey. Eventually, 800 Japanese undergraduate university students participated in the study *via* the internet. Approval for the study was obtained from the ethics committee of Hiroshima University. After participating, university students were recruited in August 2019, a slide explaining the study procedures and background was presented. Then, candidates who provided informed consent to participate pressed a button presented on the slide to respond to some questionnaire items. Only those who agreed to participate in the study responded to the questionnaire.

### Measures

#### Japanese Version of the Valuing Questionnaire

Smout et al. (2014) developed the Valuing Questionnaire (VQ) and demonstrated its reliability and validity (Doi et al., 2017). The original VQ comprises two subscales and 10 items rated on a five-point Likert-type scale (0, not at all; 6, extremely; Smout et al., 2014). Its two subscales include the Valuing Questionnaire – Progress (VQ-Progress) and Valuing Questionnaire – Obstruction (VQ-Obstruction). Reportedly, VQ-Progress reflects enactment of values, including clear awareness of what is personally important, and perseverance. Consequently, VQ-Progress reflects value-based behaviors. An example statement of VQ-Progress is “I worked toward my goals even if I did not feel motivated to do so.” By contrast, VQ-Obstruction reflects a disruption of valued living because of avoidance of unwanted experiences and distraction from values by inattention to values or attention to other psychological experiences. An example statement of VQ-Obstruction is “I spent too much time thinking about the past or future, rather than being engaged in activities that mattered to me.” Doi et al. (2017) developed and demonstrated the reliability and validity of the Japanese version of the VQ. The purpose of this study was examination of the relation between value-based behaviors and self-compassion considering the effects of some behavioral characteristics. Therefore, we used only VQ-Progress for this study to examine value-based behavior. The alpha coefficient for VQ-Progress was 0.88 in this study.

#### Japanese Version of the Behavioral Activation for Depression Scale

Kanter et al. (2007) developed the original Behavioral Activation for Depression Scale (BADS) and demonstrated its reliability and validity (Takagaki et al., 2013). The original BADS comprises four subscales and 25 items rated on a seven-point Likert-type scale (0, not at all; 6, completely; Kanter et al., 2007). Its four subscales include the Activation subscale (BADS-AC), Avoidance/Rumination subscale (BADS-AR), Work/School Impairment subscale (BADS-WS), and Social Impairment (BADS-SI). An example statement of BADS-AC is “I was an active person and accomplished the goals I set out to do.” An example statement of BADS-AR is “I did things to avoid feeling sadness or other painful emotions.” An example statement of BADS-WS is “I stayed in bed for too long even though I had things to do.” An example statement of BADS-SI is “I did things to cut myself off from other people.” Takagaki et al. (2013) developed and demonstrated the reliability and validity of the Japanese version of BADS. We used only BADS-AC and BADS-AR for this study to examine behavioral characteristics. The alpha coefficients for BADS-AC and BADS-AR used for this study were 0.84 and 0.90.

#### Japanese Version of the Short Version of the Self-Compassion Scale

Raes et al. (2011) developed the first original short version of the Self-Compassion Scale (SCS-SF), demonstrating its reliability and validity (Tomimura et al., 2012). The original SCS-SF comprises four subscales and 12 items rated on a five-point Likert-type scale (1, almost never; 5, almost always; Raes et al., 2011). Its six subscales include self-kindness, common humanity, mindfulness, self-judgment, isolation, and over-identification. An example statement of self-kindness is “Understanding of aspects I do not like.” An example statement of common humanity is “Feelings shared by most people.” An example statement of mindfulness is “Balanced view of situation.” An example statement of self-judgment is “Judgmental about my own flaws.” An example statement of isolation is “Other people are happier than I am.” An example statement of over-identification is “Fixate on everything that is wrong.” The Japanese version of the SCS-SF has good reliability and validity (Tomimura et al., 2012). We used six subscales in this study to assess relations between behavior characteristics and six factors of self-compassion. The respective alpha coefficients for SCS – self-kindness, SCS – common humanity, SCS – mindfulness, SCS – self-judgment, SCS – isolation, and SCS over-identification were 0.58, 0.63, 0.74, 0.75, 0.69, and 0.73.

#### Japanese Version of the Environmental Reward Observation Scale

Armento and Hopko (2007) developed the original Environmental Reward Observation Scale (EROS) and demonstrated its reliability and validity (Kunisato et al., 2011). The original EROS comprises 10 items that are scored on a four-point Likert-type scale (1, strongly disagree; 4, strongly agree; Armento and Hopko, 2007). An example statement of EROS is “Many activities in my life are pleasurable.” The Japanese version of EROS, which has good reliability and validity (Kunisato et al., 2011), was used to measure exposure to environmental rewards deemed necessary for increasing response-contingent positive reinforcement. The alpha coefficient for EROS used for this study was 0.76.

#### Japanese Version of Center for Epidemiologic Studies Depression Scale

Radloff (1977) developed the original Center for Epidemiologic Studies Depression Scale (CES-D) and demonstrated its reliability and validity (Shima et al., 1985). The original CES-D comprises 20 items that are scored on a four-point scale [1, rarely or never (less than 1day); 4, most or all the time (5–7days); Radloff, 1977]. An example statement is “I fee1 depressed.” The Japanese version of the CES-D has good reliability and validity (Shima et al., 1985). It was used to measure depressive symptoms. The alpha coefficient for EROS used for this study was 0.88.

### Statistical Analysis

First, this report presents the descriptive data and results of correlation analysis. Second, we divided participants into three groups based on the mean score and on the standard deviation of the VQ score: the high score group (VQ score ≧ mean+1SD), the middle score group (mean−1SD<VQ score<mean+1SD), and the low score group (≦ mean−1SD). Based on the frequency of the value-based behaviors, we used one-way MANOVA to examine differences in characteristics between behavior factors and self-compassion. Next, we applied stepwise regression analysis to assess relations among VQ-Progress (dependent), behavioral characteristics (independent variables), and self-compassion (independent variables) to examine whether each component of self-compassion is related to value-based behaviors considering the effects of some behavioral characteristics. As the first step, behavioral characteristics were examined. Finally, self-compassion factors were examined. We used software (SPSS22; SPSS Japan Inc.) for these statistical analyses.

## Results

### Descriptive Statistics and Correlation Analysis

Table 1 presents mean scores of the respective factors. We applied correlation analysis to evaluate relations among the value-based behaviors and other factors. The results demonstrated that the value-based behavior scores are positively related with those of activation (*r*=0.58, *p*<0.01), positive reinforcement (*r*=0.51, *p*<0.01), self-kindness (*r*=0.51, *p*<0.01), common humanity (*r*=0.34, *p*<0.01), mindfulness (*r*=0.49, *p*<0.01), self-judgment (*r*=0.08, *p*<0.05), and the SCS-total score (*r*=0.40, *p*<0.01; Table 1). Moreover, the value-based behavior scores were found to be related negatively with CES-D scores (*r*=−0.27, *p*<0.01).

**Table 1:** Results of descriptive data and correlation analysis.

S. No.		1	2	3	4	5	6	7	8	9		10	Mean (SD)
1.	VQ-Progress												13.92 (6.26)
2.	BADS-AC	0.58^**^											18.02 (7.49)
3.	BADS-AR	0.03	0.31^**^										15.28 (9.94)
4.	EROS	0.51^**^	0.33^**^	−0.42^**^									25.79 (5.02)
5.	SCS – Self-kindness	0.51^**^	0.39^**^	0.14^**^	0.24^**^								5.50 (1.75)
6.	SCS – Common humanity	0.34^**^	0.25^**^	0.26^**^	0.02	0.54^**^							5.32 (1.86)
7.	SCS – Mindfulness	0.49^**^	0.40^**^	0.16^**^	0.19^**^	0.65^**^	0.51^**^						5.90 (1.87)
8.	SCS – Self-judgment	0.08^*^	0.12^**^	0.45^**^	−0.32^**^	0.22^**^	0.41^**^	0.29^**^					5.46 (1.99)
9.	SCS – Isolation	0.02	0.05	0.42^**^	−0.39^**^	0.27^**^	0.35^**^	0.30^**^	0.62^**^				5.83 (2.07)
10.	SCS – Over-identification	0.02	0.07	0.42^**^	−0.37^**^	0.27^**^	0.33^**^	0.31^**^	0.61^**^	0.72^**^			6.13 (2.14)
11.	SCS-Total	0.40^**^	0.26^**^	−0.30^**^	0.56^**^	0.44^**^	0.28^**^	0.39^**^	−0.53^**^	−0.57^**^	−0.58^**^		35.30 (5.48)
12.	CES-D	−0.27^**^	−0.11^**^	0.53^**^	−0.59^**^	−0.12^**^	0.09^**^	−0.09^*^	0.37^**^	0.35^**^	0.30^**^	−0.42^**^	16.44 (9.60)

*VQ-Progress, Valuing Questionnaire-Progress; BADS-AC, Behavioral Activation for Depression Scale-Activation; BADS-AR, Behavioral Activation for Depression Scale-Avoidance/Rumination; EROS, Environmental Reward Observation Scale; SCS-Self-kindness, short version of the Self-Compassion Scale-Self-Kindness; SCS-Common humanity, short version of the Self-Compassion Scale-Common humanity; SCS-Mindfulness, short version of the Self-Compassion Scale-Mindfulness; SCS-Self-judgment, short version of the Self-Compassion Scale-Self-judgment; SCS-Isolation, short version of the Self-Compassion Scale-Isolation; SCS-Over-identification, short version of the Self-Compassion Scale-Over-identification; CES-D, Center for Epidemiologic Studies Depression Scale*.

* *p<0.05*;

** *p<0.01*.

### Differences in Behavioral Characteristics Depending on the Degree of Value-Based Behaviors

The mean score of value-based behaviors was 13.92±6.26. To examine whether behavioral characteristics differ depending on the frequency of the value-based behaviors, we classified participants into a low-value-based behavior group (value-based behavior score ≦ 7), a middle-value-based behavior group (8 ≦ value-based behavior score ≦ 20), and a high-value-based behavior group (value-based behavior score ≧ 21). We then conducted one-way MANOVA among the low-value-based behavior group, middle-value-based behavior group, and high-value-based behavior group based on the study’s dependent variables. With the use of Wilk’s criterion, a significant multivariate main effect was found [*F*(20, 1,576)=29.56, *p*<0.01]. Significant differences were found for activation [*F*(2, 797)=143.75, *p*<0.01], avoidance [F(2, 797)=5.26, *p*<0.01], positive reinforcement [F (2, 797)=112.76, *p*<0.01], self-kindness [F(2, 797)=111.43, *p*<0.01], common humanity [F(2, 797)=45.91, *p*<0.01], mindfulness [F(2, 797)=90.76, *p*<0.01], self-judgment [F(2, 797)=10.54, *p*<0.01], and depressive symptoms [F(2, 797)=28.39, *p*<0.01] among the three groups (Table 2). Next, we conducted multiple comparisons using Bonferroni method for behavioral characteristics (Table 2). From the results, we found significant differences among the three groups in BADS-AC, EROS, SCS – Self-kindness, SCS – Common humanity, SCS – Mindfulness, and CES-D.

**Table 2:** Results of multiple comparisons.

	Low-VQ group	Middle-VQ group	High-VQ group		
	*n* =130	*n* =577	*n* =93		
	Mean (SD)	Mean (SD)	Mean (SD)	*p*	
BADS-AC	10.55 (5.33)^a^	18.59 (6.46)^b^	24.94 (7.54)^c^	*p* <0.01	a<b<c
BADS-AR	13.50 (12.47)^a^	15.98 (9.19)^b^	13.39 (10.06)^c^	*p* <0.01	a<b
EROS	22.02 (4.87)^a^	25.79 (4.28)^b^	31.08 (4.76)^c^	*p* <0.01	a<b<c
SCS – Self-kindness	3.74 (1.60)^a^	5.73 (1.49)^b^	6.53 (1.80)^c^	*p* <0.01	a<b<c
SCS – Common humanity	4.02 (2.11)^a^	5.50 (1.62)^b^	6.02 (2.06)^c^	*p* <0.01	a<b<c
SCS – Mindfulness	4.35 (2.14)^a^	6.01 (1.58)^b^	7.38 (1.65)^c^	*p* <0.01	a<b<c
SCS – Self-judgment	4.85 (2.56)^a^	5.65 (1.71)^b^	5.10 (2.42)^c^	*p* <0.01	a<b, c<b
SCS – Isolation	5.64 (2.87)^a^	5.92 (1.79)^b^	5.52 (2.27)^c^	*p* =0.11	
SCS – Over-identification	5.91 (3.01)^a^	6.23 (1.82)^b^	5.76 (2.48)^c^	*p* =0.07	
CES-D	20.85 (10.72)^a^	16.26 (9.17)^b^	11.41 (7.72)^c^	*p* <0.01	c<b<a

*VQ-Progress, Valuing Questionnaire-Progress; BADS-AC, Behavioral Activation for Depression Scale-Activation; BADS-AR, Behavioral Activation for Depression Scale-Avoidance/Rumination; EROS, Environmental Reward Observation Scale; SCS-Self-kindness, short version of the Self-Compassion Scale-Self-Kindness; SCS-Common humanity, short version of the Self-Compassion Scale-Common humanity; SCS-Mindfulness, short version of the Self-Compassion Scale-Mindfulness; SCS-Self-judgment, short version of the Self-Compassion Scale-Self-judgment; SCS-Isolation, short version of the Self-Compassion Scale-Isolation; SCS-Over-identification, short version of the Self-Compassion Scale-Over-identification; CES-D, Center for Epidemiologic Studies Depression Scale*.

*Significant difference was inferred for different superscript alphabet (a vs. b vs. c; p<0.05)*.

### Results of Stepwise Regression Analysis

We applied stepwise regression analysis to assess whether each component of self-compassion was related to value-based behaviors, considering the effects of some behavioral characteristics. Stepwise regression analysis was applied using value-based behavior scores as dependent variables, and using activation, positive reinforcement, self-kindness, common humanity, mindfulness, isolation, and over-identification. As the first step, behavioral characteristics were entered. Finally, self-compassions were entered.

Results of the model were found to be significant in the first step [*F*(3, 796)=216.46, R2=0.45, p<0.01]. Activation (*β*=0.43, *t*=13.31, *p*<0.01) and positive reinforcement (*β*=0.39, *t*=11.46, *p*<0.01) were found to be significant effects, but avoidance (*β*=0.06, *t*=1.91, *p*=0.06) was not a significant effect. Results indicate significance of the model in the second step [*F*(9, 790)=104.40, R2=0.54, *p*<0.01]. Significant effects were found for activation (*β*=0.33, *t*=10.50, *p*<0.01), positive reinforcement (*β*=0.34, *t*=10.02, *p*<0.01), self-kindness (*β*=0.16, *t*=4.47, *p*<0.01), mindfulness (*β*=0.14, *t*=4.15, *p*<0.01), and common humanity (*β*=0.08, *t*=2.63, *p*<0.01). However, no significant effect was found for avoidance (*β*=−0.01, *t*=0.33, *p*=0.74), self-judgment (*β*=0.07, *t*=1.94, *p*=0.05), isolation (*β*=0.00, *t*=0.12, *p*=0.91), or over-identification (*β*=−0.03, *t*=0.77, *p*=0.44).

## Discussion

The primary purpose of this study was assessment of whether significant differences exist in some behavioral characteristics and self-compassion by the degree of the value-based behaviors. The secondary purpose of this study was examination of whether components of self-compassion are related to value-based behaviors when considering effects of behavioral characteristics. Results indicate that participants who acted in accordance with the value were more active, had more fun in daily life, and possessed characteristics of self-kindness, common humanity, mindfulness, and self-judgment. Furthermore, as stated in the hypothesis, characteristics of self-kindness, common humanity, and mindfulness were found to be related to value-based behaviors when considering the effects of behavioral characteristics.

Along with results obtained from this study, a novel finding is that positive components of self-compassion (self-kindness, common humanity, and mindfulness) are related to value-based behaviors, even when behavioral factors are considered in the context of behavioral activation. Self-kindness entails being gentle, supportive, and understanding toward oneself, rather than harshly judging oneself for shortcomings. The self is offered warmth and acceptance (Neff et al., 2019). Common humanity involves recognizing shared human experience, understanding that all humans fail, make mistakes, and lead imperfect lives (Neff et al., 2019). Mindfulness involves being aware of one’s present moment experience of adversity with clarity and balance, without running away with a dramatic storyline about negative aspects of oneself or one’s life experience (Neff et al., 2019). An earlier study yielded results suggesting that increased self-compassion is related to lower levels of depressive symptoms and anxiety (MacBeth and Gumley, 2012). The Self-Compassion program for Japanese people has been shown to engender improvement of self-compassion, anxiety, and depression compared to a control group in Japan (Arimitsu, 2016). Furthermore, a short self-compassion program conducted with university students and college students revealed increased self-compassion and reduction in anxiety and depression (Smeets et al., 2014; Dundas et al., 2017). Behavioral activation specifically assesses behavioral patterns and increases the activation of rewarding behaviors based on a person’s own values (Kanter et al., 2009; Martell et al., 2010). Acting in accordance with a person’s own values sometimes demands challenging oneself in situations that might be aversive situations. People exhibiting self-compassion buffer themselves against negative feelings during distressing social events and show moderate reactions to distressing situations (Leary et al., 2007). Treating oneself kindly despite difficulties and shortfalls allows people who are high in self-compassion to accept responsibility and to continue with other activities (Allen and Leary, 2010). Although this study is purely observational, results suggest that self-compassion might have had a role in promoting value-based behavior in the context of behavioral activation. Future studies must be conducted to develop a new program that adds a simple component of self-compassion to behavioral activation programs and which examines its effectiveness.

Comparison of behavioral characteristics and self-compassion has revealed that participants who act with medium frequency according to values had higher avoidance and self-judgment scores than others. Results of earlier studies suggest that engaging in value-based behaviors invariably triggers some form of psychological characteristic that can act as a barrier to action when the content is negative (Hayes et al., 2011). Moreover, unpleasant thoughts and emotions might arise and might become obstacles to committed action (Bear and Nagy, 2017). In a state of low frequency in value-based behaviors, one might not behave according to one’s own values. A person might begin to behave according to values in a state of mid-frequency in terms of value-based behaviors. Consequently, as reported from earlier studies (Hayes et al., 2011; Bear and Nagy, 2017), people who act with medium frequency according to values might show higher scores of avoidance and self-judgment than people in a state of low frequency in value-based behaviors.

This study has four limitations. First, this study was designed to elucidate differences in behavioral characteristics and self-compassion based on the degree of value-based behavior. Although we examined relations with behavioral characteristics using stepwise regression analysis, we did not conduct intervention with this study. Furthermore, the putative causal relations must be interpreted cautiously. Therefore, these results do not show conclusively whether self-compassion affects value-based behaviors just as activation and positive reinforcement do. Future studies must be undertaken with intervention, longitudinal research, and different experimental design to elucidate relations among behavioral characteristics. Secondly, the only participants in this study were undergraduate students. Generalizing these results to the general population can be expected to require examination of the general population in further studies. Thirdly, the Cronbach alpha of self-kindness found from this study was 0.58. The Cronbach alpha of self-kindness in the original version was 0.54 (Raes et al., 2011). The value of self-kindness found from this study is somewhat small, but it is almost equal to that of the original version. Future studies must be conducted investigate the reliability by measuring the test-retest reliability. Fourthly, because few reports have described examinations of relations among value-based behaviors and self-compassion in a behavioral activation context, this report is the first of a study elucidating the relation between value-based behaviors and self-compassion. However, this study was unable to examine the dynamics among all variables. Future studies must examine relations of some variables using structural equation modeling. The limitations described above notwithstanding, these study results suggest underlying data that promote value-based behavior in the context of behavioral activation.

For this study, we considered the effects of behavioral characteristics in behavioral activation to ascertain whether self-compassion affects value-based behaviors. This study has yielded important results that lead us to infer that self-compassion is related to value-based behaviors in the context of behavioral activation.

## Data Availability Statement

The datasets generated for this study will not be made publicly available as the authors do not have permission to share the data. Any questions regarding the data in this study should be directed to the corresponding author. Requests to access the datasets should be directed to koki.takagaki@gmail.com.

## Ethics Statement

The studies involving human participants were reviewed and approved by the ethics committee of Hiroshima University. The patients/participants provided their informed consent to participate in this study. Candidates who provided informed consent to participate pressed a button presented on the slide to respond to some questionnaire items. Only those who agreed to participate in the study responded to the questionnaire.

## Author Contributions

KT, SY, and KK conceived and designed the experiments and contributed to the writing of the manuscript and revisions. All authors approved the final article for submission.

## Funding

This study was supported by JSPS KAKENHI 19K14449.

## Conflict of Interest

The authors declare that the research was conducted in the absence of any commercial or financial relationships that could be construed as a potential conflict of interest.

## Publisher’s Note

All claims expressed in this article are solely those of the authors and do not necessarily represent those of their affiliated organizations, or those of the publisher, the editors and the reviewers. Any product that may be evaluated in this article, or claim that may be made by its manufacturer, is not guaranteed or endorsed by the publisher.
